# Shared decision-making in healthcare: development and assessment of the translated Finnish version of the SDM-Q-9

**DOI:** 10.1177/14034948241255181

**Published:** 2024-08-01

**Authors:** Milla Rosenlund, Tuuli Turja, Kaija Saranto, Hanna Kuusisto, Virpi Jylhä

**Affiliations:** 1 Faculty of Social Sciences and Business Studies, Department of Health and Social Management, University of Eastern Finland, Kuopio, Finland; 2 Faculty of Social Sciences, Tampere University, Tampere, Pirkanmaa, Finland; 3 Tampere University Hospital, Department of Neurology, Tampere, Finland; 4 The Finnish Centre for Evidence-Based Health Care: A JBI Centre of Excellence, Helsinki, Finland; 5 Research Centre for Nursing Science and Social and Health Management, Kuopio University Hospital, Wellbeing Services County of North Savo, Finland

**Keywords:** Shared decision-making, patient participation, patient experience, SDM-Q-9

## Abstract

**Aims:**

The aim of this study was to assess the cultural validity and reliability of a Finnish version of the nine-item Shared Decision-Making Questionnaire (SDM-Q-9) in a sample of patients with different sociodemographic characteristics.

**Method:**

The original SDM-Q-9 was translated into Finnish with the agreement of the developers of the original scale. The standardised translation procedure was followed by a pilot test of the questionnaire. The data were collected from an online questionnaire. Reliability was estimated by Cronbach’s alpha. Structural validity of the questionnaire was assessed by confirmatory factor analysis.

**Results:**

The pilot study assessing the cultural validity of the scale was a success, as it did not find any expressions needing to be revised. The Finnish version of the SDM-Q-9 – the SDM-Q-9-FIN – was tested in the study where a total of 736 patients responded to the questionnaire. The questionnaire yielded high reliability with a Cronbach’s alpha of 0.92. Confirmatory factor analysis confirmed the unidimensional factor structure with Item 1 excluded.

**Conclusions:**

**The SDM-Q-9-FIN was shown to be a reliable instrument for evaluating shared decision-making among Finnish patients. Further testing and research are recommended among a greater diversity of patient groups.**

## Introduction

Shared decision-making (SDM) is considered to be a process where the healthcare professional (HP) and patient are actively and equally involved in sharing their best available information and taking responsibility on making decisions concerning the care of patient [1–3]. In SDM, the HP appraises the evidence-based choices and the patient is supported in considering the options based on their values and preferences [1–4]. SDM emphasises the patient’s autonomy and right to participate in decisions about their treatment when they are aware of the available options [2]. According to previous studies, SDM has several advantages. It increases the patient’s autonomy and satisfaction [5, 6], boosts their knowledge on health, making informed decisions and participation in managing their own health [7]. SDM is additionally shown to have a positive effect on health [6], especially in disadvantaged population groups [7]. However, several factors can affect the successful realisation of SDM. These factors concern the organisation of healthcare [8], actual decision-making situations and the interaction between HP and patient [8].

There are multiple measures for assessing SDM in healthcare [9, 10]. SDM-Q-9 is a nine-item questionnaire originally developed in Germany for clinical and academic research purposes [11, 12]. The questionnaire measures the extent to which patients are involved in decision-making from the perspective of a patient [11]. The SDM-Q-9 is especially well-suited to use with preference-sensitive conditions, for which there are usually more than one suitable treatment option [5]. Although the questionnaire has been translated into several languages [13], the primary objective of this study was to develop a Finnish version of the internationally validated SDM-Q-9, and to assess its reliability and validity among Finnish patients [11, 13]. SDM-Q-9 stands out from most of the instruments due to its concise form: with standard response scales, it offers an instrument that is straightforward and easy to use. It is also convenient when conducting repeated measures in the interactive process of SDM.

## Methods

### Translation of SDM-Q-9 into Finnish

SDM-Q-9 was developed for evaluating SDM from the patient’s perspective and for clinical purposes to assess the quality of healthcare [11]. The questionnaire contains two open-ended questions and nine items concerning the SDM process at the doctor’s appointment. In our survey, the open-ended questions were left out as we had other background questions concerning the appointment. For each item, the respondent indicated the extent to which they agreed or disagreed on a 6-point scale ranging from ‘Completely disagree’ to ‘Completely agree’.

The original questionnaire was developed in German. Permission for its translation into Finnish and use was obtained from the core development team at University Medical Center Hamburg-Eppendorf in Germany. The translation procedure followed the instructions of the core development team. First, two translators independently translated the questionnaires from German to Finnish. These two translations were discussed jointly in a multidisciplinary research group, and a primary consensus version was attained. Then, the primary consensus version was independently translated back into German by a third translator. The third translator was not familiar with the original version. All three were authorised translators. The back translation was then sent to the core development team for feedback. The back translation received only minor comments, which related to cultural appropriateness such as using the expression ‘the doctor’ instead of what would have been a direct translation, ‘my doctor’, as in Finland a family doctor system is not in use. Both SDM-Q-9 and SDM-Q-Doc (the physician version of the SDM-Q-9) were translated in the process, but only SDM-Q-9 was used in this study.

The items in the SDM-Q-9 were originally scaled on six points, ranging from ‘Completely disagree’ to ‘Completely agree’. In our survey, the scale was changed to a 4-point scale to make the difference between scale points semantically clearer to Finnish respondents. Choices ‘Strongly disagree’ and ‘Strongly agree’ were left out. The scale thus ranged from ‘Completely disagree’ (= 1) to ‘Completely agree’ (= 4). The total score, calculated by summing the score of the nine items, could range between 9 and 36. Permission for the scale change was granted by the SDM-Q-9 developers. The translation in Finnish follows the original 6-point scale. All questions concerning the healthcare appointment and SDM-Q-9 were set as mandatory fields in the online questionnaire.

### Pilot testing of cultural and face validity

After completion of the translation procedure, the face validity of the items of the Finnish version of the questionnaire were assessed in two focus groups. One group consisted of researchers (*n* = 5) from multidisciplinary fields (e.g. medicine, health and human services informatics and social psychology), some of them with clinical expertise. The other group consisted of a sample of patients who had recently had a doctor’s appointment (*n* = 9) with different sociodemographic backgrounds (e.g. age, education, health status). The participants were not familiar with the questionnaire beforehand. The items were also assessed in regard to cultural validity, which here refers to those sociocultural influences that shape the way respondents made sense of the items semantically and linguistically [14]. The participants were able to give feedback on each item. No comments were given concerning the linguistic or semantic translation of the SDM-Q-9. The respondents had only minor comments that did not necessitate modification of the questionnaire. The Finnish translation of the SDM-Q-9 is presented in Supplementary file 1.

## Study design

### Data collection

Between December 2021 and January 2022, an online questionnaire was administered to assess patients’ and citizens’ experiences of and motivation to participate in SDM. The survey was targeted at members of the Finnish Pensioners’ Federation (ca. 120,000) and the Finnish Neuro Society (ca. 10,000 members), which were chosen because of the high number of members and their characteristics (i.e. elderly people and patients with long-term illness). Later the survey was given to the Finnish Epilepsy Association (ca. 10,000 members) and the Organisation for Respiratory Health in Finland (ca. 25,000 members). The invitation to participate in the survey was sent directly to members of the Finnish Pensioners’ Federation via email addresses from the member database (*N* = 30,329). All the organisations shared the link of the survey in their social media accounts (Facebook and Twitter) and official website. The Finnish Neuro Society additionally shared the link in their newsletter. In the analysis, the Finnish Pensioners’ Federation is considered as one group and the respondents from the patient associations as one collective group (Finnish Neuro Society, Finnish Epilepsy Association and the Organisation for Respiratory Health in Finland).

A total of 736 respondents completed the SDM-Q-9. The study complies with the regulations of the Finnish Advisory Board of Research Integrity and, more broadly, with the World Medical Association Declaration of Helsinki. The respondents were informed about the study, use of the data for the research, data protection and storage, and rights for using the data. Before answering the survey, the respondents were asked for their consent to participate in the research. The anonymity, confidentiality and informed consent of the respondents were maintained while gathering and handling the data. No direct identifiers of respondents were gathered in the survey. The data maintenance cycle followed the data management principles of the PROSHADE project funded by the Strategic Research Council within the Academy of Finland.

### Survey items

In the survey respondents’ background information concerning their health status, use of health services and opinions about use and utility of digital health services was acquired. Respondents were also asked whether they had had an appointment with a doctor and a decision concerning the treatment had been made within the past 6 weeks [15]. If the patient had had an appointment with a doctor, the SDM-Q-9 was made available for collecting answers. In cases where a respondent had not had an appointment with a doctor, the questionnaire was not visible to the respondent. Respondents who had had an appointment were asked further questions: 1) What was the reason for the appointment? 2) Was the appointment a first visit or a follow-up visit? 3) Was the doctor a general practitioner, specialist or occupational health physician? 4) Was the appointment face to face, a telephone or video/virtual visit? At the end, the questionnaire also included a few background questions concerning sociodemographic characteristics.

### Data analysis

Item analysis on the SDM-Q-9 was carried out, describing the frequencies, percentages, means and standard deviation (SD) for each item as well as for the total scores. Reliability was measured in a variety of ways. Firstly, Cronbach’s alpha was used for measuring the internal consistency of responses. Cronbach’s alpha was reported as it is regularly used for measuring the reliability of a questionnaire: a value from 0.7 to 0.95 is considered to provide good evidence of internal consistency [16]. According to previous studies [e.g. 11], we hypothesised that the internal consistency would reach a Cronbach’s alpha value of >0.9. Secondly, corrected total item correlations and means for corrected total item correlations were shown. Finally, the individual inter-item correlations, range of inter-item correlations and means for inter-item correlation were measured.

Confirmatory factor analysis (CFA) was conducted to assess the structural validity of the questionnaire [17, 18, 19]. Based on previous studies [11, 20, 21] the unidimensional factor structure was tested with three models. In Model 1 all nine items were included to test the unidimensional factor structure [11]. In Model 2, Item 1 was excluded based on the study by Hulbæk et al. [20]. For Model 3, Items 1 and 9 were excluded based on the results from Rodenburg-Vandenbussche et al.’s research [21]. The results of the chi-square test (χ^2^), comparative fit index (CFI), root mean square error of approximation (RMSEA) and the standardised root mean square residual (SRMR) are reported to indicate whether the data fit Models 1 to 3. Values >0.90 for CFI and ⩽0.050 for RMSEA and SRMR were considered desirable [22]. Additionally, factor loadings and explained variances (*h*^2^) of the models were reported.

Statistical analyses were all performed using IBM SPSS Statistics (version 27). IBM SPSS Amos (version 27) was used for CFA.

The open-ended answers concerning the reason for the appointment were categorised into three categories according to the medical issue of concern. The categories were treatment of long-term illness, treatment or diagnosis of symptom/illness/disease, or preventive treatment/follow-up.

## Results

### Characteristics of the respondents

A total of 736 respondents who had had a doctor’s appointment during the previous 6 weeks answered the SDM-Q-9. The mean age of the respondents in the total sample was 68.35 (SD = 10.01). The majority of the respondents were female (64%) and 40% of the respondents had at least high school or vocational education and 42% had a bachelor’s degree as their highest degree. Sociodemographic characteristics of the respondents and information on appointments are presented in Table I.

**Table I.** Sociodemographic characteristics of the respondents and information on appointments.

Count % (*n*)	Finnish Pensioners’ Federation 85% (629)	Patient associations 15% (107)	Of the total (*N* = 736)
Gender			
Women	60 (378)	86 (94)	64 (472)
Men	40 (251)	14 (13)	36 (264)
Other	0	0	0
Age			
Mean (years)	71	52	68
Education			
Elementary school or similar	14 (88)	4 (4)	13 (92)
High school or vocational education	26 (164)	36 (39)	27 (203)
Bachelor’s degree	44 (273)	32 (34)	42 (307)
Master’s degree	15 (94)	23 (25)	16 (119)
Doctoral or licentiate degree	1 (10)	5 (5)	2 (15)
Subjective health status			
Good	15 (95)	3 (3)	13 (98)
Fairly good	41 (259)	23 (25)	39 (284)
Average	35 (216)	40 (43)	35 (259)
Fairly poor	9 (59)	28 (30)	12 (89)
Poor	0	16 (6)	1 (6)
Reason for the appointment			
Long-term illness (e.g. medication, follow-up)	39 (246)	64 (69)	43 (315)
Diagnosing the symptom/disease	57 (360)	33 (35)	54 (395)
Preventive care	3 (21)	3 (3)	3 (24)
Other reason	<1	<1	<1 (2)
Purpose of the appointment			
First appointment	44 (273)	26 (28)	41 (301)
Follow-up	33 (207)	30 (32)	32 (239)
Periodic follow-up for long-term illness	24 (148)	44 (47)	26 (195)
Not known			1 (1)
Did you visit . . .			
General practitioner	52 (328)	23 (25)	48 (353)
Specialist	66 (70)	66 (70)	49 (363)
Occupational physician	11 (12)	11 (12)	3 (20)
Location of the appointment			
Face to face	93 (587)	79 (84)	91 (671)
Phone call	6 (40)	19 (20)	8 (60)
Video or other distance connection	<1 (2)	2 (3)	<1 (5)

Most respondents felt that their health status was average or fairly good (74%). The reason for the respondents’ last appointment was mainly long-term illness and its treatment (43%) or diagnosis of a symptom or disease (54%). Despite the COVID-19 pandemic, which occurred during the time of the survey (December 2021 to January 2022), nearly all appointments took place face to face (91%).

### Descriptive analysis of the SDM-Q-9 items and reliability analysis

**Figure 1.**
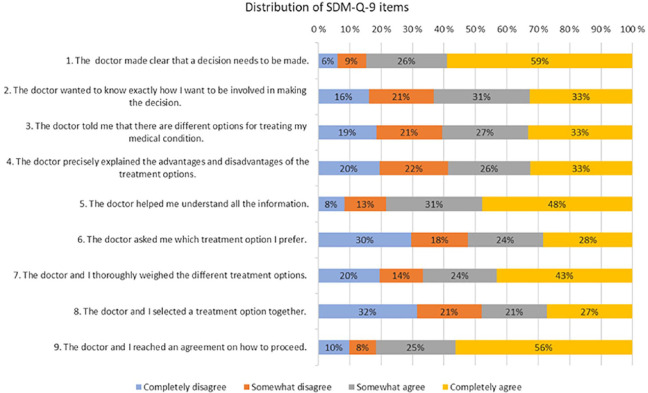
The distribution of each SDM-Q-9 item for all respondents.

The total score for the Finnish version of SDM-Q-9 (SDM-Q-9-FIN) could in this study range between 9 and 36. In the total sample, mean SDM total score (SD) was 25.96 (7.59). Respondents from the Finnish Pensioners’ Federation had scores above the mean, 26.38 (7.56), contrary to respondents from the patient associations, 24.14 (6.92). Among men, the total mean score (SD), 26.27 (7.19), was slightly higher than among women, 25.78 (7.80). Supplementary file 2 shows the aggregate means for SDM for various groups. For single items, the total item mean score for SDM-Q-9 items ranged between 2.44 (Item 7) and 3.38 (Item 1), while the overall item mean score was 2.88. Figure 1 presents the distribution of single items for all respondents.

The overall total mean score was higher among respondents from the Finnish Pensioners’ Foundation (2.93) than among respondents from the patient associations (2.76). The overall total mean score for men (2.92) was slightly higher than for women (2.80). Supplementary file 3 presents the single item mean scores in each respondent group.

### Reliability

The SDM-Q-9 provided good reliability with a Cronbach’s alpha of 0.92. The Cronbach’s alpha when a specific item is deleted is shown in Table II. Corrected item total correlations ranged from 0.537 (Item 1) to 0.796 (Item 6) (Table II). The mean value for corrected item total correlations was 0.71. Values for inter-item correlations ranged from 0.366 (between Items 1 and 7) to 0.813 (between Items 3 and 4) (Supplementary file 4). The mean for inter-item correlations was 0.56, which suggests that items were strongly correlated.

**Table II.** Corrected item total correlations.

Item	Corrected item total correlations	Cronbach’s alpha if item deleted
1. The doctor made it clear that a decision needed to be made.	0.537	0.92
2. The doctor wanted to know exactly how I wanted to be involved in making the decision.	0.748	0.91
3. The doctor told me that there were different options for treating my medical condition.	0.695	0.91
4. The doctor precisely explained the advantages and disadvantages of the treatment options.	0.795	0.90
5. The doctor helped me understand all the information.	0.744	0.91
6. The doctor asked me which treatment option I preferred.	0.796	0.90
7. The doctor and I thoroughly weighed the different treatment options.	0.794	0.90
8. The doctor and I selected a treatment option together.	0.746	0.91
9. The doctor and I reached an agreement on how to proceed.	0.599	0.92

### Factor structure

CFA was conducted using three different models based on the results of previous studies [11, 22, 23]. Model 1 aimed to confirm the unidimensional structure of the SDM-Q-9 [11]. In Model 2, Item 1 was excluded [22] and in Model 3 Items 1 and 9 were excluded [23]. All models met the criteria for CFI. For SRMR, Models 2 and 3 met the criteria. The CFA results are given in Table III. Factor loadings and explained variances for the items of the models are presented in Table IV.

**Table III.** Results of the confirmatory factor analysis.

Model	χ^2^	CFI	SRMR	RMSEA
1	434,952	0.905	0.0531	0.143
2	323,5	0.923	0.0469	0.144
3	216.91	0.943	0.0374	0.140

**Table IV.** Factor loadings and explained variances (*h*^2^) of the items for Models 1 to 3.

Item	Model 1		Model 2		Model 3	
	Factor loading	*h* ^2^	Factor loading	*h* ^2^	Factor loading	*h* ^2^
1. The doctor made it clear that a decision needed to be made.	0.541	0.293	-	-	-	-
2. The doctor wanted to know exactly how I wanted to be involved in making the decision.	0.768	0.589	0.757	0.573	0.751	0.565
3. The doctor told me that there were different options for treating my medical condition.	0.740	0.547	0.740	0.547	0.744	0.554
4. The doctor precisely explained the advantages and disadvantages of the treatment options.	0.833	0.693	0.829	0.687	0.833	0.694
5. The doctor helped me understand all the information.	0.753	0.567	0.740	0.547	0.728	0.529
6. The doctor asked me which treatment option I preferred.	0.855	0.730	0.865	0.748	0.872	0.761
7. The doctor and I thoroughly weighed the different treatment options.	0.852	0.726	0.864	0.746	0.869	0.755
8. The doctor and I selected a treatment option together.	0.771	0.595	0.770	0.59	0.757	0.573
9. The doctor and I reached an agreement on how to proceed.	0.608	0.369	0.601	0.361	-	-

- indicates that the item was not included in the model.

## Discussion

This study aimed to translate the Finnish version of the SDM-Q-9, SDM-Q-9-FIN, and to assess its validity and reliability. To ensure content and semantic accuracy, the translation procedure followed the instructions of the questionnaire’s developers [11]. The translation was assessed in regard to face- and cultural validity from both linguistic and semantic perspectives.

In the study, 736 individuals completed the questionnaire. The results of the SDM-Q-9-FIN questionnaire indicated that the scale is a reliable tool for measuring the degree of SDM. Cronbach’s alpha (0.92) showed high internal consistency for corrected item total correlations. The results of the analysis were in line with other studies measuring the reliability of the SDM-Q-9 [11, 22–28]. Reliability measures did not waver in the examination of deleted items. Hence, the results confirmed that the nine-item total formed a consistent scale for measuring SDM.

For the single items, mean score ranged between 2.44 (Item 7) and 3.38 (Item 1). The overall item mean score was 2.88. The results can be interpreted as, for most patients, it was obvious that a decision had to be made (Item 1). However, patients did not feel that they were not asked which treatment option they preferred (Item 7). For the whole sample mean, an SDM total score (SD) of 25.96 (7.59) was detected. Variations among different patient groups still existed.

In this study, CFA was conducted presuming a unidimensional structure, as observed in the original study of Kriston et al. [11] and in the results of the studies by Hulbæk et al. [20] and Rodenburg-Vandenbussche et al. [21]. Our results confirmed the unidimensional structure and the structural validity of SDM-Q-9-FIN. All models met the criteria for CFI and Models 2 and 3 also met the criteria for SRMR, indicating good structural validity. CFA did not quite confirm the one-factor structure, as none of the models met the criteria for RMSEA in regard to the fixed cutoffs [22]. This has been noted in other studies [e.g. 24, 28] and indicates that dynamic fit index cutoffs should be considered instead of fixed cutoffs [22, 29]. Nevertheless, all items showed adequate factor loadings ranging from 0.541 (Item 1) to 0.855 (Item 6). Item 1 showed the lowest correlation to total score and factor loading (0.541), which is consistent with other studies [20, 24].

## Strengths and limitations

The major strength of this study lies in its implementation of the SDM-Q-9-FIN. With its nine-item content, the questionnaire is of a convenient length for respondents. In this study, an adequate sample size with over 100 respondents per item was attained [30]. The main limitation of the study concerns the sample. The study was targeted at specific organisations’ members, however, ultimately the majority of the respondents were pensioners (85%) and thus elderly people. A sample with a wider age range and different health backgrounds would have been favourable, allowing exploration of differences between the subsamples. Research involving different kinds of patient groups is needed, despite the results being consistent with previous studies among different nationalities and health statuses [11, 23–28] In the study, a 4-point scale was used instead of the original 6-point scale, which did not seem to impact the consistency of the results in comparison to other studies.

## Conclusions

The results of the study suggest that the SDM-Q-9-FIN is an appropriate questionnaire for measuring the degree of SDM in healthcare. Translating the SDM-Q-9 into Finnish was a straightforward, quite effortless process. In a pilot study, the translated scale showed excellent cultural validity, which was followed by assessments of reliability and structural validity with convincing results. The high internal consistency among the items of the SDM-Q-9-FIN indicates that the scale is a recommendable tool in studies of SDM in healthcare.

Further research among different patient groups is needed to afford better insights concerning SDM in different contexts in Finnish healthcare.

## Supplemental Material

sj-docx-1-sjp-10.1177_14034948241255181 – Supplemental material for Shared decision-making in healthcare: development and assessment of the translated Finnish version of the SDM-Q-9 Supplemental material, sj-docx-1-sjp-10.1177_14034948241255181 for Shared decision-making in healthcare: development and assessment of the translated Finnish version of the SDM-Q-9 by Milla Rosenlund, Tuuli Turja, Kaija Saranto, Hanna Kuusisto and Virpi Jylhä in Scandinavian Journal of Public Health

sj-docx-2-sjp-10.1177_14034948241255181 – Supplemental material for Shared decision-making in healthcare: development and assessment of the translated Finnish version of the SDM-Q-9 Supplemental material, sj-docx-2-sjp-10.1177_14034948241255181 for Shared decision-making in healthcare: development and assessment of the translated Finnish version of the SDM-Q-9 by Milla Rosenlund, Tuuli Turja, Kaija Saranto, Hanna Kuusisto and Virpi Jylhä in Scandinavian Journal of Public Health

sj-docx-3-sjp-10.1177_14034948241255181 – Supplemental material for Shared decision-making in healthcare: development and assessment of the translated Finnish version of the SDM-Q-9 Supplemental material, sj-docx-3-sjp-10.1177_14034948241255181 for Shared decision-making in healthcare: development and assessment of the translated Finnish version of the SDM-Q-9 by Milla Rosenlund, Tuuli Turja, Kaija Saranto, Hanna Kuusisto and Virpi Jylhä in Scandinavian Journal of Public Health

sj-docx-4-sjp-10.1177_14034948241255181 – Supplemental material for Shared decision-making in healthcare: development and assessment of the translated Finnish version of the SDM-Q-9 Supplemental material, sj-docx-4-sjp-10.1177_14034948241255181 for Shared decision-making in healthcare: development and assessment of the translated Finnish version of the SDM-Q-9 by Milla Rosenlund, Tuuli Turja, Kaija Saranto, Hanna Kuusisto and Virpi Jylhä in Scandinavian Journal of Public Health
