# Supplementary material for: Shared decision-making in healthcare: development and assessment of the translated Finnish version of the SDM-Q-9
Source: Scand J Public Health. 2024 Aug 1;53(7):713–20. doi: 10.1177/14034948241255181 (PMC12598053; doi:10.1177/14034948241255181)
Supplement: sj-docx-2-sjp-10.1177_14034948241255181 – Supplemental material for Shared decision-making in healthcare: development and assessment of the translated Finnish version of the SDM-Q-9 [file sj-docx-2-sjp-10.1177_14034948241255181.docx]

Supplementary file 2

Aggregate means for SDM scores in various groups

| **Patient group** | SDM Mean (SD) |
| --- | --- |
| Finnish Pensioners’ Federation  Patient associations  Total | 26.38 (7.56)  24.14 (6.92)  25.96 (7.59) |
| **Gender**  Women  Men | 25.78 (7.80)  26.27 (7.19) |
| **Education**  Elementary school or similar  High school or vocational education  Bachelor’s degree  Master’s degree  Doctoral or licentiate degree | 26.84 (7.51)  26.15 (7.48)  26.03 (7.48)  24.46 (7.68)  28.47 (6.38) |
