# Supplementary material for: Shared decision-making in healthcare: development and assessment of the translated Finnish version of the SDM-Q-9
Source: Scand J Public Health. 2024 Aug 1;53(7):713–20. doi: 10.1177/14034948241255181 (PMC12598053; doi:10.1177/14034948241255181)
Supplement: sj-docx-4-sjp-10.1177_14034948241255181 – Supplemental material for Shared decision-making in healthcare: development and assessment of the translated Finnish version of the SDM-Q-9 [file sj-docx-4-sjp-10.1177_14034948241255181.docx]

Supplementary file 4

Inter-item correlations

|  | 1. The doctor made clear that a decision needs to be made. | 2. The doctor wanted to know exactly how I want to be involved in making the decision. | 3. The doctor told me that there are different options for treating my medical condition. | 4. The doctor precisely explained the advantages and disadvantages of the treatment options. | 5. The doctor helped me understand all the information. | 6. The doctor asked me which treatment option I prefer. | 7. The doctor and I thoroughly weighed the different treatment options. | 8. The doctor and I selected a treatment option together. | 9. The doctor and I reached an agreement on how to proceed. |
| --- | --- | --- | --- | --- | --- | --- | --- | --- | --- |
| 1. The doctor made clear that a decision needs to be made. |  | 0.529 | 0.388 | 0.468 | 0.544 | 0.383 | 0.366 | 0.425 | 0.389 |
| 2. The doctor wanted to know exactly how I want to be involved in making the decision. | 0.529 |  | 0.549 | 0.645 | 0.604 | 0.647 | 0.608 | 0.607 | 0.496 |
| 3. The doctor told me that there are different options for treating my medical condition. | 0.529 | 0.549 |  | 0.720 | 0.530 | 0.632 | 0.628 | 0.501 | 0.397 |
| 4. The doctor precisely explained the advantages and disadvantages of the treatment options. | 0.468 | 0.645 | 0.720 |  | 0.659 | 0.701 | 0.695 | 0.582 | 0.447 |
| 5. The doctor helped me understand all the information. | 0.544 | 0.604 | 0.530 | 0.659 |  | 0.588 | 0.590 | 0.604 | 0.558 |
| 6. The doctor asked me which treatment option I prefer. | 0.383 | 0.647 | 0.632 | 0.701 | 0.588 |  | 0.813 | 0.656 | 0.468 |
| 7. The doctor and I thoroughly weighed the different treatment options. | 0.366 | 0.608 | 0.628 | 0.695 | 0.590 | 0.813 |  | 0.687 | 0.485 |
| 8. The doctor and I selected a treatment option together. | 0.425 | 0.607 | 0.501 | 0.582 | 0.604 | 0.656 | 0.687 |  | 0.594 |
| 9. The doctor and I reached an agreement on how to proceed. | 0.389 | 0.496 | 0.397 | 0.447 | 0.558 | 0.468 | 0.485 | 0.594 |  |
